# Impact of Soil and Water Conservation Measures on Soil Physicochemical Characteristics and Crop Productivity: Evidence From Korekore Watershed in the Central Highlands of Ethiopia

**DOI:** 10.1155/tswj/2679180

**Published:** 2024-12-23

**Authors:** Abera Fantahun, Tesfaye Mebrate Lemma, Fikrey Tesfay, Yitea Seneshaw Getahun

**Affiliations:** ^1^Gishe Rabel District Office of Agriculture, North Shewa Zone, Debre Brihan, Amhara National Regional State, Ethiopia; ^2^Department of Natural Resource Management, College of Agriculture and Natural Resource Sciences, Debre Berhan University, Debre Birhan, Ethiopia

**Keywords:** crop yield rate, cultivated land, soil and water conservation, soil degradation, soil degradation index

## Abstract

This study investigates the effects of physical and chemical soil degradation on crop productivity in the *Korekore* Watershed. The watershed was categorized into level, sloping, and steep slope gradients. Within each slope gradient, one cultivated land managed with soil and water conservation and one cultivated land without soil and water conservation were selected. Selected soil physicochemical analyses were performed on 18 disturbed and undisturbed soil samples and were taken from the top 0–20 cm depths from each cultivated land with three replications. The soil degradation index (SDI) was evaluated based on the percent changes in soil properties of cultivated land without soil and water conservation to that of the cultivated land managed with soil and water conservation. The crop productivity index was determined by analyzing the percentage growth rate and its standard deviation. Soil bulk density and moisture content improved undercultivated land managed with SWC practices. Significantly higher (*p* ≤ 0.05) soil organic carbon (SOC) content (2.02%), total N (0.12%), available P (4.69 ppm), and exchangeable K (1.33 cmolc/kg) were observed in cultivated land managed with SWC as compared to cultivated land without SWC measures. The value of the SDI (+243.9%) for the watershed has demonstrated that soil degradation has improved and soil and water conservation measures had a significant impact on crop yields. The average crop yield rate (CYR) (30%) was found to be lower than the estimate provided by the central statistics agency in Ethiopia. A higher CYR was observed in cultivated land managed with soil and water conservation practices. It can be concluded that physical and chemical soil degradation significantly impacts crops unless counterbalanced by soil and water conservation measures. Therefore, it is recommended to implement well-integrated watershed management practices to improve soil quality and enhance crop productivity.

## 1. Introduction

Soil degradation refers to the loss of soil's capacity to provide ecosystem services due to the reduction in soil productivity and quality [1–3]. Globally, soil degradation is dramatically increasing, and it poses a tremendous challenge to increase agricultural productivity, as well as threatening the livelihoods of people [4, 5]. Africa in particular is the most vulnerable and severely affected region to land degradation and desertification [6], which poses a threat to both survival on this planet and national prosperity [7]. Ethiopia is among the sub-Saharan belt countries facing land degradation with soil erosion being the major that results in the depletion of nutrients and fertility [8].

Studies have showed that land degradation in the Ethiopian highlands is attributed to several complex factors of biophysical, socioeconomic, and policy factors [9–13]. Especially, Ethiopia has severe issues with soil erosion and degradation, especially in the highland regions where topography (steep-slope) is the primary cause of degraded land surfaces [14, 15]. Improper use and management of soil resources also continue to be the main causes of soil degradation [5, 16, 17]. Moreover, population pressure, soil, climate, deforestation, unmanaged exploitation of resources, and land management are also the predominant factors that promote degradation revealed to reduce crop yield in Ethiopia [18–21]. The current severity and extent of soil degradation pose the most significant threat to food security in the country [22, 23]. Common types of soil degradation in the central highlands of Ethiopia include soil erosion, acidification, solidification, salinization, compaction, and reduction of organic matter and vegetation degradation [2, 24, 25].

Ethiopia's ability to sustain agricultural production is still seriously threatened by soil degradation that is most frequently due to water-induced soil erosion [22, 26]. As a consequence, in the highlands of Ethiopia, the average soil loss from croplands is estimated to be between 130 and170 t·ha^−1^·yr^−1^ [22, 27] and also can reach the range of 300–400 tons·ha^−1^·yr^−1^ depending on the surface slope, land cover, and rainfall intensities [26, 28]. This represents approximately up to 10–13 mm of soil depth loss annually [29]. However, the erosion rate for croplands in the entire Ethiopian highlands was estimated to be 35 t·ha^−1^·yr^−1^ [30] and increased to 42 t·ha^−1^·yr^−1^ [26]. Because of conservation efforts, the soil loss from croplands was reduced to 36.8 t·ha^−1^·yr^−1^ [31]. Plot-level measurements in the Ethiopian highlands have also confirmed that soil erosion ranges from 0 to 170 t·ha^−1^·yr^−1^ [32]. A review by the authors in [33] has showed that the highest soil erosion rate was observed in the moist zone which is 57 ± 7.8 t·ha^−1^·yr^−1^.

A synthesis analysis by the authors in [30] has showed that the highest soil erosion rates next to the moist zone were measured in subhumid areas with less variable high rainfall and intensive cultivation. Studies conducted by the authors in [34] have showed that due to land degradation associated with soil erosion, soil nutrients are depleted in Ethiopia. The authors in [35] had quantified the nutrient loss to reach 30 kg of N·ha^−1^, and 15–20 kg P ha^−1^ nutrients are depleted annually in Ethiopia. According to the authors in [36], total N loss ranged from 39.6 to 55.5 kg·ha^−1^·yr^−1^, whereas available P loss ranged from 4.1 to 5.9 kg·ha^−1^·yr^−1^. Similar previous studies by the authors in [37, 38] have also showed that sustainable land management practices have reduced the loss of total N and available P associated with water by 48% and 54%, and by 58% and 66%, respectively.

The effect of soil erosion when revealed in productivity loss leads to a significant annual cost measured at about US$ 4.3 billion [18]. The above findings indicated that erosion reduces crop productivity, which in turn leads to a decline in the degree of erosion control due to decreasing vegetative cover [39]. The immediate impact of soil degradation is the reduction of crop yield and productivity [40]. Longer term effects of rill erosion include decreased soil depth, water-holding capacity, fertility, organic matter content, and cation exchange capacity (CEC). These factors ultimately result in decreased crop yield and decreased vegetation growth [22]. The challenge of soil degradation is not far from the *Gishe Rable* District, particularly in the *Korekore* Watershed, which is part of the Ethiopian highlands. Due to water erosion, intensive farming on steep slopes, and a lack of conservation measures, soil degradation in this area is at a severe stage.

In most central highland parts of Ethiopia, efforts have been made to implement various but dominantly physical soil and water conservation measures since the 1970s through public campaigns and with the support of different programs. Particularly, in the *Gishe Rable* District as part of the massive campaign, farmers have implemented several physical SWC measures in the early 1990s. The constructed physical SWC measures are mixes of different types and purposes which are located in different parts of the watershed. Initially, the major focus was on constructing stone and soil bunds, but later on, establishing physical conservations with the support of vegetative measures became part of the planning and implementation. Bunds are small embankment-type structures, constructed across the land slope, either with soil or stone to reduce the slope length, resulting in diminished runoff, reduced soil erosion, and enhanced soil infiltration [41]. Rainwater harvesting and smaller moisture conservation structures, such as microbasins and trenches, were also integrated some years later after the soil-backed stone bunds were constructed.

Although the sustainability of the implemented SWC measures was overlooked, governmental and nongovernmental organization–initiated efforts to extend watershed-based integrated conservation are continued in many parts of the country. Besides, watershed communities are cumulating experiences through targeted planning and implementation of measures toward controlling and reversing the degradation processes. Using the lessons from the variety of efforts, for instance, the Ethiopian Government has promised to restore more than 15 million ha as part of its African Forest Landscape Restoration Initiative (AFR100) commitment [42]. Participatory forest management, area exclosures, and sustainable land management program and the Green Legacy Initiative (SLM-GLI) initiatives have been used to achieve this target [43].

Although various types of SWC measures are implemented, their sustainability and effectiveness are often questioned. The gap could be manifested by the little attention given to study the impact of the cumulative effects and long-term impacts of the intervened SWC measures on cultivated lands and their production variables. Most previous studies have focused primarily on specific SWC measures [44–48], which may not fully reflect the reality on the ground with respect to the types and distribution of measures. In many areas of the country where SWC practices have been implemented over different years, cultivated land is significantly positively affected, at least due to the presence of physical SWC independently and support from agronomic conservation measures. Reduced soil erosion due to slow down of water run-off and soil stabilization and consequent fertility of topsoil are some of the significant effects of physical SWC measures on cultivated lands. While it is essential to examine the impact of individual SWC measures on cultivated land, investigating the collective impact of mixed types and irregularly constructed SWC is much closer to the reality on the ground. Therefore, assessing whether there is a change in soil properties on cultivated land due to these SWC measures is crucial. Understanding the association between soil degradation and crop yield is critical to assess the negative impact of soil degradation, and consecutively to evaluate the benefits of soil conservation measures. This suggests that watershed–scale-specific data on soil erosion is required to support timely data for decision-makers and land managers to have proactive soil conservation strategies and development plans [49].

A study focusing on the evaluation of soil degradation and its effects on selected crop productivity using different indexes is crucial in response to soil degradation through the recommendation of appropriate SWC measures. Soil degradation index (SDI) is one of the indexes to quantify the degree of soil degradation in a given area. SDI is a tool used to assess the health and quality of soils which involves evaluating various environmental factors. Besides assessing the extent and severity of soil degradation, the SDI is used for effective monitoring and management of soil resources. It also highlights the complexity of soil degradation processes, necessitating tailored approaches for different regions and conditions. It is based on the assumption that the status of the soil properties under cultivated land managed with SWC is less disturbed than the status of the soil properties under cultivated land without SWC [50]. Consequently, this study was conducted to (1) examine soil physicochemical properties of cultivated land under different conservation measures and (2) determine the level of soil degradation and crop productivity status on selected cultivated land.

## 2. Materials and Methods

### 2.1. Study Area

**Figure 1:**
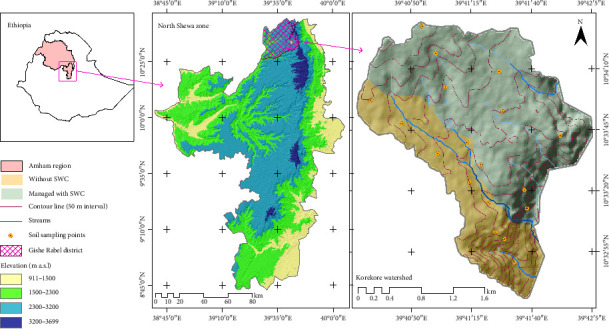
Location map of the study area.

**Figure 2:**
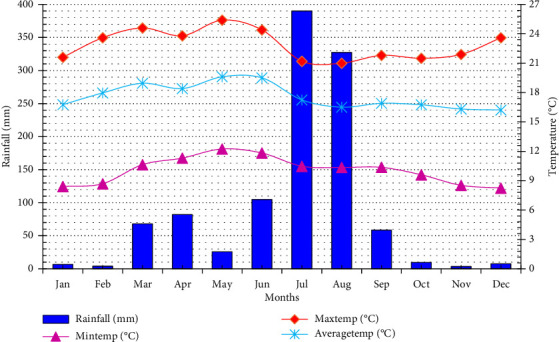
Long-term average of monthly rainfall and temperature (1990–2021).

The study was conducted in the *Korekore* Watershed, which is located 375 km northeast of Addis Ababa on the eastern edge of the Ethiopian highlands. The watershed is in the *Gishe Rable* District of the North Shewa Zone, which is part of the Amhara National Regional State (Figure 1). The watershed is stretched between the latitude of 10° 34′ 28″ N to 10° 32′ 36″ N and longitudes of 39° 40′ 28″ E to 39° 42′ 4″ E geographic coordinates. A 30-year (1990–2021) climate data obtained from the Ethiopian National Meteorological Institute indicated that the monthly highest rainfall is recorded in July and August (268 mm per month). As indicated in Figure 2, September and June come next, with monthly rainfall rates of 113 mm and 186 mm, respectively. The month of May set a record for the highest monthly maximum, minimum, and average temperatures (25°C, 12°C, and 17°C, respectively). The lowest minimum temperature was indicated in January (8.40°C) and December (8.20°C). Dominantly, the *Korekore* Watershed is characterized by a moist highland climate. The topography of the area is rugged, mountainous, and dissected by a number of rivers, streams, and gullies. The region's bottom landscapes are prone to flooding. Geologically, the *Korekore* Watershed is dominantly covered by Tarmaber Megezez formation of the middle Miocene geological epoch: transitional and alkaline basalts rock types [51]. The soil types of the area are Eutric Cambisols, Eutric Regosols, Lithosols, and rock surfaces, which cover 269 ha (52%), 142 ha (27%), 54 ha (10%), and 54 ha (10.4%), respectively [52]. Wheat (*Triticum aestivum*) and barley (*Hordeum vulgare*) are the major cereal crops grown widely in the area. Bean (*Vicia faba* L.), one of the pulses cultivated in the area, also plays an important role in crop rotation. Agriculture which integrates mixed farming, both crop production and livestock rearing, is the main economic activity and livelihood strategy for the people in the *Korekore* Watershed. The watershed covers a total area of 519 ha. From the total area, about 374 and 23 ha are covered by cultivated and shrubland, respectively. The rest 122 ha is bare degraded land. According to the Gishe Rable District agricultural office, the average landholding size of the study area is about 0.75 ha per household.

### 2.2. Experimental Design and Soil Sampling

**Figure 3:**
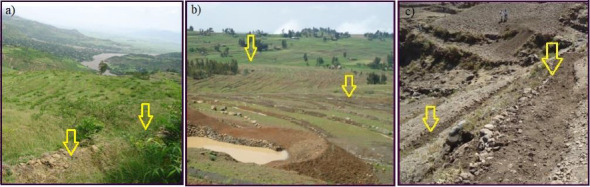
Partial view of different SWC measures in the *Korekore* Watershed. (a) Stone bund supported with animal forage, (b) water collection trenches, and (c) stone bund displacement.

The *Korekore* Watershed was selected purposefully based on its representation of both cultivated lands managed with soil and water conservation measures and without SWC practices. The cultivated land managed with SWC was managed by different physical SWC structures (soil bund, stone bund, trench, and terrace) and agronomic conservation measures (tree lucerne, grass, and tree plantation) since 1990s (Figure 3). Since then, different SWC measures have been applied annually on the watershed, and most of the time the agronomic conservation measures serve as stabilization of the physical SWC measures. On the contrary, cultivated land without SWC was not managed by integrated physical and agronomic SWC practices. The sampled sites managed with SWC and without SWC land were found nearby to each other to keep other factors constant. First, the watershed was categorized into three slope gradient steep (> 30%), sloping (15%–30%), and level (0%–15%) slope categories following the first level hierarchy of major landforms [53]. Within each slope gradient, two cultivated land-use practices (one cultivated land managed with SWC and one cultivated land without SWC) were selected. Each cultivated land in each slope gradient has three replications.

Soil samples were collected from each of the purposefully selected cultivated land-use practices from the top 0–20 cm depth in October 2019, which is a drier season (Figure 2). Approximately, 1 kg of composite soil samples was prepared by collecting soil samples from five points with a zigzag layout. The entire field can be sampled using a zigzag pattern, which will result in recommendations for soil tests that are more consistent [54]. To analyze dry bulk density and soil moisture content (SMC), additional undisturbed cylindrical soil core samples (at a depth of 0–20 cm) were taken from the same locations where the composite soil samples were collected. A total of 18 composite soil samples (2 cultivated, i.e., managed with SWC measures and without SWC practices *X* 3 slope gradients and *X* 3 replications) composite soil samples were collected. The soil samples underwent air-drying at 25°C (room temperature) and were sieved through various mesh sizes as per the requirements for analyzing soil properties [55].

### 2.3. Soil Laboratory Analysis

Soil particle size analysis was done by the Bouyoucos hydrometer method [56], and the USDA soil texture triangle was used to determine the textural class. For soil bulk density, the core sample method was used [57], and SMC was determined using the gravimetric method [55]. Soil pH was measured by 1:2.5 soils: water suspension using a pH meter [58]. Electrical conductivity (EC) was analyzed in 1:5 soil: water suspension using an EC meter [59]. Soil organic carbon (SOC) was determined by the Walkley–Black oxidation method [60]. Total nitrogen (N) was analyzed by using the Kjeldahl digestion method [61]. Available phosphorus (P) was analyzed by using the Olsen method which samples were extracted with a sodium bicarbonate solution of pH 8.5, and reading measure absorbance was performed using a spectrophotometer [62]. A titrimetric method by distillation of ammonia that was displaced by sodium was used to estimate the CEC of the sample soils [63]. Exchangeable cations (Na and K) were determined from the extraction of 1 M ammonium acetate (NH_4_OAc) solution buffered at pH 7.0 and was read using flame photometry [64]. Soil laboratory analysis was conducted in Debre Birhan Agricultural Research Center Soil Laboratory in Debre Birhan, Ethiopia.

### 2.4. Soil Degradation Index Determination

SDI is one of the indexes to quantify the degree of soil degradation in a given area. It is possible to qualify soil properties deterioration by computing the soil nutrients contents and/or properties under different degradation status [65, 66]. The SDI was computed based on the assumption that the status of the soil properties under cultivated land managed with SWC is less disturbed than the status of the soil properties under cultivated land without SWC. It is assumed that the status of soil properties under the cultivated land was once similar to less disturbed forestland [66]. Accordingly, the SDI indicates the percent changes in soil properties of cultivated land without SWC compared to that of the cultivated land managed with SWC [67]. In this study, the levels of soil degradation in each type of land management were indicated by the calculated values of the SDI of the selected soil parameters. The SDI was computed by considering the values of soil parameters (factors that affect plant growth): SOC, total N, available P, exchangeable K, and bulk density. A widely applied equation was used to calculate the SDI of individual soil properties/parameter [65–67] (as shown in the following equation).

(1) $$\begin{array}{c} \text{SDI}\left(\%\right)=\frac{X\text{with SWC}-X\text{without SWC}}{X\text{without SWC}}\times 100, \end{array}$$

where SDI is the soil degradation index and *X* is the a soil parameter (SOC, total N, available P, K, and bulk density) mean value. To quantify the cumulative SDI, first the difference between the individual soil properties was calculated and expressed in percentage. Then, the cumulative of the percentage of the computed difference of the selected soil properties were used to quantify the SDI of the watershed. A cumulative (total) positive value of the SDI indicates an overall positive improvement in individual soil properties. In case, where a negative change of the SDI in individual soil property is considered as an improvement in soil productivity, a negative SDI value of a soil property can sum up to a positive cumulative (total) SDI. The cumulative negative value of the SDI shows the intensity of soil degradation, and the more negative values correspond to more degraded soils [65, 68]. If the cumulative of individual soil properties' SDI values sum up to zero, then it shows a balance in degradation in the agricultural ecosystem. Degradation cannot be zero; however, there is always a balance. If a zero cumulative SDI appears, it indicates a balance between the deterioration of some soil properties and the improvement of other soil properties. Studies conducted by the authors in [67, 68] have employed similar techniques to calculate the cumulative SDI.

### 2.5. Crop Yield Rate (CYR) Determination

To assess the impact of SWC measures on crop yield, an index of crop yield was used. The crop yield index is a measure used to assess the productivity of a crop in terms of the amount of produce harvested per unit area. It helps in assessing the effectiveness of different farming techniques. It also evaluates how changes in environmental conditions or management practices affect crop productivity. The CYR percentage for this study was calculated using the following equation:

(2) $$\begin{array}{c} \text{Growth Rate Percentage}=\frac{\text{Yield in Year }X-\text{Yield in Year }X-1}{\text{Yield in Year }X-1}\times 100, \end{array}$$

where Yield in Year *X* is the wheat yield weight for a specific year and Yield in Year *X* − 1 is the wheat yield weight for the previous year. Wheat (*Triticum aestivum* L.) yield data in tons per hectare for 5 years (2015–2019) were obtained from the Gishe Rable District Office of Agriculture. In the case of our study, the most reliable data on crop production from individual farmers' cultivated land can only be accessed from the district agricultural office through the agricultural agents in the Kebele (the smallest administrative structure in Ethiopia). Only a single dominantly cultivated variety of durum wheat was considered for this study purpose. Wheat crop yield trends for 5 years were analyzed.

### 2.6. Statistical Analysis

Prior to any statistical analysis, the soil data were subjected to skewness and kurtosis tests to assess normality and homogeneity. The general linear model (GLM) procedure with a two-way analysis of variance (ANOVA) was used to statistically analyze the soil data that were obtained from the soil sample using SAS 9.2 software. Mean separation was performed for treatments that showed statistically significant differences (*p* < 0.05) using Tukey's honest significant difference (HSD) test.

## 3. Results and Discussion

### 3.1. Soil Physical Properties

Clay and silt fractions differed significantly (*p* ≤ 0.05) between cultivated lands managed with and without SWC measures. Cultivated lands managed with SWC had a significantly higher mean value (48.7%) of clay content (Table 1). Conversely, significantly higher sand content (43.8%) was recorded under cultivated land without SWC measures. The presence of SWC measures such as trench, stone and soil bunds, and tree plantation may have resulted in higher clay particle content, which is effective at restoring soil physicochemical properties [44]. Related findings were stated by the authors in [69] who observed significantly higher clay content under bunded farmland in the Aba Gerima catchment, Blue Nile basin, Ethiopia. Soils under cultivated land managed with SWC were dominated by clay, whereas soils under cultivated land without SWC measures were more influenced by sandy clay loam soil texture [70]. This justifies the selective nature of soil erosion between fine and coarse soil particles, in that when finer particles are removed by erosion, coarser particles increase in proportion, leading to a higher proportion of finer particles in the deposited area [71].

The level slope exhibited a statistically significant (*p* ≤ 0.01) higher mean value of clay (58.3%). On the other hand, the sloping recorded lower silt content (18.3%). This could be because finer materials such as clay were transported further and deposited on the level slope position than coarse materials. Clay particles can transport longer distances in suspension than silt and sand particles [72]. The slope is an important factor in determining erosion and sediment transport processes [73–75] as different soils have varying particle resistance to detachment and susceptibility to transportation [76]. Sandy soils are less cohesive than clayey soils; thus, aggregates with high sand content are more easily detached. Slope gradient and cultivated land management interaction effects were not statistically significant (*p* > 0.05) (Table 1).

Soil bulk density was significantly (*p* ≤ 0.05) varied between cultivated lands managed with and without SWC measures. The mean bulk density was significantly higher (1.27 kg·m^−3^) under cultivated land without SWC measures (Table 1). Removal of crop residues [77] and continuous cultivation [78, 79] can cause soil compaction, which could explain the higher bulk density observed under cultivated land without SWC measures. The significantly higher sand content in the cultivated land without SWC conservation is a reason for the higher bulk. Soil bulk density and soil clay content have a negative relationship; soils with higher clay content tend to have lower bulk density [80]. Livestock trampling during overgrazing [81] in cultivated land without SWC could also be an additional factor for the high bulk density in the cultivated land without SWC. According to the [80] classification system, the soil bulk density under both cultivated land-use practices falls within the range for recently cultivated soils that do not restrict plant root growth.

SMC significantly differed (*p* ≤ 0.01) among cultivated lands managed with SWC and those without. The mean SMC was significantly higher (30.6%) in cultivated land managed with SWC (Table 1). The physical and agronomic conservation practices (i.e., crop residue and tree plantation) SWC measures applied could increase water infiltration and explain the higher SMC in the cultivated land managed with SWC. A study conducted in the Dawnt watershed, northwestern Ethiopia, by the authors in [82], showed similar results with significantly high SMC under cultivated land managed with SWC. Moreover, the significantly higher clay content in the cultivated lands (Table 1) could also contribute to the increased SMC in the cultivated lands managed with SWC. Based on a similar study by the authors in [83], a higher soil capacity to hold water and nutrients is indicated by higher clay content. SMC varied significantly (*p* < 0.01) along the slope gradient (Table 1), with higher values of SMC (28.9%) recorded in gentle slope areas. This variation may be due to the natural internal gravitational flow of subsurface flow to the lower geographically lesser slope gradient areas. This difference may be also attributed to the SWC measures implemented in the steep slope areas, which affected water-influenced water infiltration and the storage capacity of the slope gradient within the study watershed [82].

### 3.2. Soil Chemical Properties

#### 3.2.1. Soil pH and EC

The soil pH value was not significantly (*p* > 0.05) influenced by the SWC measures applied to the cultivated land (Table 2). Since there was a numerical difference but no statistical difference, according to the soil pH rating [84], the soil pH of both the cultivated land managed with SWC and without SWC was rated as slightly acidic. However, the soil pH value significantly varied (*p* < 0.05) along the slope gradient (Table 2). A significantly high soil pH value (6.70) was recorded on the sloping gradient (15%–30%), whereas the lowest soil pH value (6.58) was found in the steep slope gradient (> 30%). A similar study by the authors in [85] in the Ele Watershed in southern Ethiopia reported high soil pH for the sloping class and low soil pH for the steep slope. Due to increased soil erosion, leaching, and a decrease in soluble base cations caused by steeper slopes and high rainfall, the soil pH in the steep slope was significantly lower, which resulted in higher H^+^ activity. In a similar vein, a research result by the authors in [86] explained that the leaching and runoff resulting from accelerated erosion cause the loss of base-forming cations, which in turn lowers soil pH and contributes to increased soil acidity. The buildup of exchangeable bases on the level sloping and sloping gradients may be the cause of the high soil pH there [85]. The interaction effect between slope gradient and cultivated land management is statistically insignificant (*p* > 0.05) (Table 2). The soil EC in both the cultivated land managed with SWC and without SWC was rated as salt-free [80], and there was no statistical difference (*p* > 0.05) between cultivated land management practices and slope gradient.

#### 3.2.2. SOC and Total Nitrogen

Significantly higher (*p* ≤ 0.05) SOC content was observed in cultivated land managed with SWC (2.02) as compared to cultivated land without SWC measures (Table 2). Higher SOC content in cultivated land managed with SWC is believed be due to integrated soil physical and agronomic conservation practices used in the watershed. A similar study carried out by the authors in [82] in the Dawnt Watershed, Central Gondar, Ethiopia, recorded higher SOC content under areas managed with SWC compared to areas without management. A study conducted in Old Goa, India, by the authors in [87] also showed that the implementation of SWC measures on sloping cultivated land has a significant potential to improve the SOC. SOC has significantly (*p* ≤ 0.05) varied along slope gradients (Table 2). Significantly, the highest value of SOC (2.51%) was found in soils of steep slope gradient, and the lowest SOC content (1.13%) was recorded in soils of sloping areas. The highest SOC was found due to the vegetation type that was applied to support the SWC measures in the steep slope gradient of the watershed. This was justified by the authors in [88] that poor land management and frequent cultivation of the land result in low returns of soil improving organic inputs which promote the depletion of SOC. This suggests that the implementation of SWC measures on the hillside within the study area has a significant influence on enhancing the SOC levels. The SOC in cultivated land managed with SWC measures was moderate, whereas the SOC in the cultivated land without SWC was rated as low according to the authors in [84]. Land management which has bunds stabilized by vegetation and is free from free grazing has higher SOC levels than that of the lands not managed with SWC [82]. The interaction effect of cultivated land management and slope gradient did not show statistically significant differences (*p* > 0.05) in SOC undermanaged with SWC and without SWC (Table 2).

Applying SWC measures significantly (*p* ≤ 0.05) affects the total N content of the soils (Table 2). The mean value of the total N was higher in cultivated land managed with SWC measures (0.12%) and lowest in the soils without SWC measures in the watershed (0.08%). The physical SWC measures in the area might be the cause for the higher soil total N content in the cultivated land managed with SWC measures. A study [89] revealed that the mean total N content of the managed sites was higher compared with adjacent sites without SWC. However, according to the authors in [80], the soil total N under both cultivated land managed with SWC and without SWC measures was rated as low. The low rate of soil total N in both cultivated lands managed with SWC and without SWC may be attributed to the management of legume crops and crop residue. The integration of legume plant species with physical SWC measures will significantly improve the total N content of degraded cultivated lands [12, 47, 90]. The effectiveness of physical SWC measures in improving soil total N depends on the type, scale, and management of the integration of agronomic conservation measures and legume crop management [12]. Crop residue management is one major factor that affects the soil total N status on cultivated lands [45, 91]. Thus, the low rate of soil total N in the study watershed's cultivated lands might be related to crop residue management. Although agronomic conservation measures being observed along contour, gullies, and steep slope areas, the cultivated land lacks continued crop rotation using legume crops, and crop residue is completely harvested. The *Korekore* Watershed is known for its dominant cultivation of wheat and barley crops. Therefore, the result of this study suggests that to significantly improve the soil total N on degraded cultivated land, the integration of legume plant species and crop residue management is crucial. The interaction effect of cultivated land management (SWC) and slope gradient does not show any significant differences (*p* > 0.05) in soil total N (Table 2).

#### 3.2.3. Available Phosphorus

The available P contents of the soils were significantly (*p* ≤ 0.01) influenced by the applied SWC measures (Table 2). Significantly higher available P was recorded under cultivated land managed with SWC (4.69 ppm). This practice shows the highest available P, indicating that SWC measures can significantly enhance available P, which is crucial for crop growth. The available P status in cultivated lands can be influenced significantly by factors such as the presence of SWC structures [92] and the type and application rate of organic and inorganic fertilizers [93]. Soil and water conservation measures can reduce the loss of phosphorus-rich soil particles and improve water infiltration, which can help to increase the availability of phosphorus to plants [92]. Phosphorus availability in the soil increases with an increase in crop residue and availability of soil microbial [94]. Thus, the reason behind the statistically significant difference in available P between the cultivated lands in the study watershed is the application of integrated SWC measures. A study conducted on highly eroded slopes of Tigray, North Ethiopia, showed an increase in available P on terrace benches than nonterraced land [48]. The results of this study are in line with the findings that similarly discovered that farm plots with soil conservation structures had significantly higher concentrations of available P compared to the neighboring nonconserved farm plots [46].

However, even though there was a statistically significant difference, the available P in all the cultivated lands was rated low, which is questionable and potentially limiting crop productivity [80]. Such a low content of available P in cultivated land occurs due to the way the soils of cultivated land are managed [95]. Phosphorus is removed from soils when crops are harvested [96]. Long-term cultivation can significantly reduce microbial biomass, which consequently reduces phosphate activities [97]. Therefore, despite the *v* in the cultivated lands with SWC, this suggests that the cultivated land management pattern in the *Korekore* Watershed should consider long-term cover that increases the abundance of P-solubilizing bacteria, which activate soil P as they improve the soil environment [95]. The available P contents of the cultivated land soils of the *Korekore* Watershed do not show any statistically significant (*p* > 0.05) difference along the slope gradient (Table 2). The interaction effect of cultivated land management and slope gradient did not show statistically significant differences (*p* > 0.05) (Table 2) on the available P of cultivated lands.

#### 3.2.4. CEC and Exchangeable K and Na

The mean value of CEC 22.2 cmolc/kg recorded both in the cultivated land managed with SWC and without measure was rated medium (Table 3) [80]. The medium CEC value of soils is probably due to the predominance of clay contents and higher SOM content in the area. Soil and water conservation influence soil CEC in a way that it rises as the percentage of clay in the soil, i.e., the finer the texture of the soil, the higher the CEC. This result is in agreement with the findings of Abebe [98] who reported a variation in CEC values because of variation in organic matter content and the amount of clay particles.

The average of CEC values was statistically different (*p* ≤ 0.05) with reference to the slope gradient (Table 3). It ranged from 16.8 cmolc/kg at the steep slope to 24.2 cmolc/kg at the level. According to the rating by Hazelton and Murphy [99], the soils of the study area have moderate (24.2–10.9 cmolc/kg) CEC values. The presence of considerable clay content and SOM in both the cultivated lands may be the cause for the moderate rate of CEC under three slope gradients. Considering the interaction effect of cultivated land management and slope gradient, there were statistically no significant differences (*p* > 0.05) in CEC (Table 3).

Exchangeable K concentration was significantly (*p* ≤ 0.05) affected by cultivated land management type (Table 3). Statistically significantly higher mean value (1.33 cmolc/kg) of exchangeable K was recorded under cultivated land managed with SWC. This might be because cultivated lands with physical SWC practices have a reduced soil erosion rate, and leaching of exchangeable K is limited due to their attachment with clay, i.e., fixation. The outcome was consistent with a study by the authors in [100], which found that cultivated lands treated with SWC had higher exchangeable K values than untreated cultivated lands. The significantly lower exchangeable K in cultivated land without the SWC measure may be caused by the increased loss of K-rich soil particles. Exchangeable K can lose due to soil erosion, and improper land management which results in low SOM, and free grazing beyond carrying capacity [101]. According to the authors in [102], exchangeable K results in the study area were categorized as moderate. Considering the interaction effect of cultivated land management and slope gradient, there were statistically no significant differences (*p* > 0.05) under cultivated land managed with SWC and without SWC measures (Table 3).

Exchangeable Na was significantly (*p* < 0.05) affected by cultivated land management (Table 3). The mean value was significantly higher under cultivated land managed with SWC (0.65 cmolc/kg). This could be associated with sediment trapped in the applied SWC measures. However, the exchangeable Na content in both cultivated lands was still significant [80]. According to the authors in [102], exchangeable Na in the study watershed was moderate. This finding suggests that cultivated lands with SWC have better soil structure and infiltration conditions than farmlands without SWC. Exchangeable Na alters the chemical and physical makeup of the soil, mainly through the swelling and dispersal of organic and clay particles, which reduces airflow and water permeability [103]. Deforestation, leaching, inadequate crop residue absorption into the soil, less fallow periods or continuous cropping, and soil erosion have all been linked to the depletion of essential cations as compared to managed SWC regions. The findings aligned with those of a study carried out in the Maybar areas of North Ethiopia's South Wello Zone [104]. This study stated that soil degradation–causing factors have collectively contributed to the reduction of essential cations in unconserved agricultural lands when compared to nearby conserved land.

### 3.3. SDI

The SDI value of the *Korekore* Watershed was +243.9% (Table 4). Of the soil properties, total N and available P have showed higher improvement in the SDI followed by Exch K+ and SOC. Bulk density and CEC have showed the lowest improvement in the SDI. It is observed that the soil properties under cultivated land managed with SWC had a higher positive change, indicating less soil degradation. The value of SDI in the *Korekore* Watershed could be a sign of the rehabilitation of deteriorated soil quality. This could be due to the application of different physical and agronomic conservation measures under cultivated land managed with SWC, such as terraces, leaving residues on the soil surface, planting trees, and grass species. This helps to increase and maintain water stored in the soil, reduces soil erosion, and contributes to reducing soil disturbance and buildup of SOM. A similar study conducted in the western part of Lithuania showed that the SDI was significantly influenced by the applied soil management techniques [105]. A study conducted by the authors in [68] in the Gumara Watershed, Lake Tana basin of Northwest Ethiopia, observed the highest deterioration index value under cultivated land than shrubland. This showed that poor land-use practices frequently resulted in soil quality degradation. Soil conservation activities can alter the physical characteristics of the soil, such as its organic matter content, soil structure, water holding capacity, bulk density, soil porosity, soil pH, and its workability [106].

### 3.4. CYR

In the *Korekore* Watershed, the analysis of the CYR percentage showed that soil degradation had an effect on crop yields (Table 5). The average annual wheat yield in the study watershed has ranged between 1.88 t·ha^−1^ in the cultivated land without SWC measure and 2.46 t·ha^−1^ in the cultivated land managed with SWC. Within the periods considered in the study, a higher CYR percentage of wheat (10%) was recorded under the cultivated land managed with SWC than the cultivated land managed without SWC (7.40%). The higher CYR percentage recorded in the cultivated land managed with SWC indicates that the applied SWC measure has increased the CYR and affected soil productivity. The observed increase in CYR is directly associated with the improvement of soil productivity which is expressed as a SDI. The significant reduction in soil erosion and associated nutrient loss due to the SWC in the watershed could be the main factor for the increased CYR percentage. Particularly, the positive change in the SDI of the most crop yield–limiting nutrients such as SOC (+38.4), total N (+50.4), and available phosphorous (+50.8) (Table 4) is an indication of improvement of the soil productivity under the SWC measures in the *Korekore* Watershed. Studies conducted in Ethiopia have showed that the positive effect of SWC measures on soil properties has increased crop productivity [107]. Though between 2015 and 2019 higher crop yield was recorded in the cultivated land managed with SWC, the CYR of this study was much lower than the estimation of the Central Statistics Agency of Ethiopia (Central Statistical Agency [108]). Overall, the finding from this study suggests that the crop yield of significantly degraded cultivated land can be positively affected through the application of integrated SWC measures.

## 4. Conclusions

This study investigated the levels of physical and chemical soil degradation and their effects on crop productivity in the *Korekore* Watershed. The results of this study revealed that the majority of the physical and chemical characteristics of soil varied significantly in reference to how the cultivated land was managed. Specifically, soil bulk density, SMC, SOC, total N, available P, CEC, and exchangeable Na and K were significantly improved under cultivated land managed with SWC practices. The SDI value for the *Korekore* Watershed was +243.9%, indicating an increase in soil quality. The outcomes showed that SWC practices have a beneficial effect on restoring the fertility of degraded lands and soil quality. This is attributed to the improvements in soil properties that were brought about by the application of SWC measures. This demonstrates that soil degradation had a significant impact on crop yields in the *Korekore* Watershed. Cultivated land managed with SWC practices showed a higher CYR, while land without SWC practices had a lower growth rate. Thus, the application of SWC measures to the highland watershed in Ethiopia resulted in a significant improvement in wheat yields. Except for soil pH and SOC content, none of the measured soil properties showed significant variation along the slope gradient. Similarly, significant interaction effects for all soil properties were not observed. A potential reason for this outcome might be the application of soil and water conservation measures which can mask the effect of slope gradient on soil properties. This could be due to reduced soil erosion and increased SMC. The relatively small watershed size, the homogeneity of soil parent materials, uniform climate type, and similar land-use practices across the watershed could also contribute to the nonsignificant interaction observed in our study. Overall, to maintain sustainable agricultural production in the watershed, integrated soil and water management is essential for improving the physicochemical characteristics of the soil for enhanced crop production and productivity indefinitely.

## Figures and Tables

**Table 1:** Mean ± S.E of soil physical properties on different cultivated land management practices and slope gradients in the *Korekore* Watershed.

Factors	Attribute of factors	Particle size distribution (%)			Texture	*ρ*b (kg·m^−3^)	Soil moisture content (%)
		Clay	Silt	Sand			
Land management practice (LMP)	M-SWC	48.7 ± 7.5^a^	27.8 ± 1.9	23.6 ± 8.6^b^	CL	0.9 ± 0.1^b^	30.6 ± 0.4^a^
	W-SWC	31.8 ± 6.2	23.8 ± 3.2	43.8 ± 9.4^a^	SCL	1.3 ± 0.1^a^	24.1 ± 0.4^b^
	*p* value	0.004	0.29	0.02		0.04	0.007

Slope gradient (SG)	0%–15%	58.3 ± 3.5^a^	30.0 ± 1.5	10.7 ± 3.4^c^	CL	1.1 ± 0.1	28.9 ± 0.6^a^
	15%–30%	18.3 ± 3.7^c^	21.7 ± 3.8	60.0 ± 7.0^a^	SL	1.0 ± 0.0	27.2 ± 0.5^a^
	> 30%	44.0 ± 8.9^b^	25.7 ± 3.6	30.3 ± 1.1.9^b^	CL	1.2 ± 0.1	25.9 ± 0.5^b^
	*p* value	< 0.001	0.22	0.001		0.31	0.006

	LMP ∗ SG	0.06^ns^	0.46^ns^	0.22^ns^		0.97^ns^	0.09^ns^

	CV (%)	24.6	29.9	49.7		8.32	2.43

*Note:* Means ± S.E. with different letters within a column are significantly different (*p* < 0.05) (Fisher's LSD). *ρ*b = soil bulk density, LMP ∗ SG interaction effect.

Abbreviations: C = clay; CL = clay loam; CV = coefficient of variation; ns = nonsignificant; SCL = sandy clay loam; S.E. = standard error; SL = sandy loam.

**Table 2:** Mean ± S.E of soil pH, EC, SOC, total N, and available P on different cultivated land management practices and slope gradients in the *Korekore* Watershed.

Factors	Attribute of factors	pH	EC (ds/m)	SOC (%)	Total N (%)	Available P (ppm)
Land management practice	M-SWC	6.8 ± 0.12	0.06 ± 0.007	2.02 ± 0.26^a^	0.12 ± 0.01^a^	4.69 ± 0.32^a^
	W-SWC	6.6 ± 0.06	0.07 ± 0.008	1.46 ± 0.24^b^	0.08 ± 0.01^b^	3.11 ± 0.31^b^
	*p* value	0.45	0.62	0.04	< 0.0001	0.003

Slope gradient	0%–15%	6.7 ± 0.01^a^	0.07 ± 0.006	1.59 ± 0.19^b^	0.10 ± 0.03	4.24 ± 0.53
	15%–30%	6.7 ± 0.05^a^	0.06 ± 0.01	1.13 ± 0.14^b^	0.09 ± 0.03	3.19 ± 0.37
	> 30%	6.6 ± 0.03^b^	0.07 ± 0.01	2.51 ± 0.32^a^	0.10 ± 0.02	4.28 ± 0.52
	*p* value	0.008	0.76	0.001	0.06	0.09

	LMP ∗ SG	0.26^ns^	0.24^ns^	0.79^ns^	0.20^ns^	0.74^ns^

	CV (%)	1.2	35.7	29.2	9.94	22.6

*Note:* Means ± S.E. with different letters within a column are significantly different (*p* < 0.05) (Fisher's LSD). LMP ∗ SG interaction effect.

Abbreviations: CV = coefficient of variation; LMP = land management practice; M-SWC = managed with soil and water conservation; ns = nonsignificant; S.E. = standard error; SG = slope gradient; W-SWC = without SWC.

**Table 3:** Mean ± S.E of soil CEC and Exch. K and Na on different cultivated land management practices and slope gradients in the *Korekore* Watershed.

Factors	Attribute of factors	CEC (cmol_c_/kg)	Exch. K^+^ (cmol_c_/kg)	Exch. Na^+^ (cmol_c_/kg)
Land management practice	M-SWC	22.2 ± 0.06	0.75 ± 0.08^a^	0.65 ± 0.04^a^
	W-SWC	17.3 ± 0.07	0.54 ± 0.04^b^	0.53 ± 0.05^b^
	*p* value	0.07	0.03	0.0002

Slope gradient	0%–15%	24.2 ± 0.07^a^	0.65 ± 0.10	0.58 ± 0.11
	15%–30%	18.2.06^a^	0.56 ± 0.05	0.60 ± 0.07
	> 30%	16.8 ± 0.07^b^	0.73 ± 0.09	0.58 ± 0.05
	*p* value	0.006	0.28	0.69

	LMP ∗ SG	0.13^ns^	0.41^ns^	0.26^ns^

	CV (%)	26.7	27.04	8.47

*Note:* Means ± S.E. with different letters within a column are significantly different (*p* < 0.05) (Fisher's LSD). LMP ∗ SG interaction effect.

Abbreviations: CV = coefficient of variation; LMP = land management practice; M-SWC = managed with soil and water conservation; ns = nonsignificant; S.E. = standard error; SG = slope gradient; W-SWC = without SWC.

**Table 4:** The soil degradation index under cultivated land managed with SWC and without SWC measures in the *Korekore* Watershed.

Soil properties	Cultivate land		SDI%
	Without SWC	Managed with SWC	
*ρ*b (kg·m^−3^)	1.27	0.90	−29.1
SOC (%)	1.46	2.02	+38.4
Total N (%)	0.08	0.12	+50.0
Available P (ppm)	3.11	4.69	+50.8
CEC (cmol_c_/kg)	17.3	22.2	+28.6
Exch. K^+^ (cmol_c_/kg)	0.51	0.75	+47.1
Cumulative SDI%			+243.9

*Note:ρ*b = bulk density.

Abbreviation: SDI = soil degradation index.

**Table 5:** Sampled percentage crop yield rate in the *Korekore* watershed.

Year	Wheat yield (tone ha^−1^)		Annual rainfall (mm)
	Managed with SWC	Without SWC	
2015	2.00	1.60	896.48
2016	2.10	1.90	1429.10
2017	2.60	1.80	1144.34
2018	2.70	2.00	1017.77
2019	2.90	2.10	1360.55
Average	2.46	1.88	
Crop yield rate percentage	10.02	7.40	
Standard deviation	0.39	0.09	

## Data Availability

The data that support the findings of this study are available from the corresponding author upon reasonable request.
